# The Effect of 4-Octyldecyloxybenzoic Acid on Liquid-Crystalline Polyurethane Composites with Triple-Shape Memory and Self-Healing Properties

**DOI:** 10.3390/ma9090792

**Published:** 2016-09-21

**Authors:** Jianfeng Ban, Linjiang Zhu, Shaojun Chen, Yiping Wang

**Affiliations:** 1 Nanshan District Key Lab for Biopolymers and Safety Evaluation, Shenzhen Key Laboratory of Polymer Science and Technology, Guangdong Research Center for Interfacial Engineering of Functional Materials, College of Materials Science and Engineering, Shenzhen University, Shenzhen 518060, China; jianfengban@gmail.com (J.B.); linjiangzhu@gmail.com (L.Z.); 2 Key Laboratory of Optoelectronic Devices and Systems of Ministry of Education and Guangdong Province, College of Optoelectronic Engineering, Shenzhen University, Shenzhen 518060, China

**Keywords:** shape memory, liquid crystalline, self-healing

## Abstract

To better understand shape memory materials and self-healing materials, a new series of liquid-crystalline shape memory polyurethane (LC-SMPU) composites, named SMPU-OOBAm, were successfully prepared by incorporating 4-octyldecyloxybenzoic acid (OOBA) into the PEG-based SMPU. The effect of OOBA on the structure, morphology, and properties of the material has been carefully investigated. The results demonstrate that SMPU-OOBAm has liquid crystalline properties, triple-shape memory properties, and self-healing properties. The incorporated OOBA promotes the crystallizability of both soft and hard segments of SMPU, and the crystallization rate of the hard segment of SMPU decreases when the OOBA-content increases. Additionally, the SMPU-OOBAm forms a two-phase separated structure (SMPU phase and OOBA phase), and it shows two-step modulus changes upon heating. Therefore, the SMPU-OOBAm exhibits triple-shape memory behavior, and the shape recovery ratio decreases with an increase in the OOBA content. Finally, SMPU-OOBAm exhibits self-healing properties. The new mechanism can be ascribed to the heating-induced “bleeding” of OOBA in the liquid crystalline state and the subsequent re-crystallization upon cooling. This successful combination of liquid crystalline properties, triple-shape memory properties, and self-healing properties make the SMPU-OOBAm composites ideal for many promising applications in smart optical devices, smart electronic devices, and smart sensors.

## 1. Introduction

Stimuli-responsive polymers play an important role in many applications, such as diagnostics, smart optical systems, and tissue engineering, as well as in micro-electromechanical systems, biosensors, textiles, and coatings [1,2]. Shape memory polymers (SMPs) are one type of important stimulus-responsive polymer that can adopt temporary shapes and recover their original shapes upon exposure to external stimuli [3,4]. Although the basic shape memory behaviours of SMPs are focused on elongation, bending, and swelling, through proper material structure and stimuli design, complicated shape memory behaviours such as walking, rotation, and vibration can also be achieved. The general mechanism of the shape memory effect (SME) is attributed to the reversible switching structure of SMPs. This reversible switching structure may be responsive to stimuli such as heat, electricity, magnetic field, light, and Ph [5,6,7]. Thus, SMPs have received the increased attention of researchers for many potential applications.

To date, numerous SMPs based on various structures have been developed, including polyurethane, polystyrene copolymer, and epoxy-based polymers. Among them, the thermal-induced shape memory polyurethane (SMPU) has been mostly used in practice because of its competitive mechanical and shape memory properties, e.g., high specific strength, high parameters of shape fixity and shape recovery, and a transition temperature that can be approximately tuned to room and body temperature [8,9]. A variety of SMPUs have been reported with a wide range of activation temperatures and applications, such as medical devices, heat shrinkable packages, mechanical actuators, sensors, and self-deployable structures. However, common SMPUs cannot satisfy the growing trend of new multi-functional materials. Recently, the important development of shape memory polymer composites (SMPCs) with different fillers has significantly broadened the varieties and functionalities of SMPs, which significantly expand their applications [10,11,12]. For example, spatially controlled electro-active shape recovery is achieved in CNT-filled SMPCs [13]. Selective light heating is reported in gold nanoparticle-filled SMPCs [14]. Multiple-SMEs have also been developed in the norbornyl-polyhedral oligomeric silsesquioxanes (POSS) copolymer [15]. These studies indicate that the investigation of SMPCs is one of the most important research directions in the field of SMPs and polymer composites. It provides a new way to prepare polymer composites with diversified shape memory properties and more functionality [16,17].

As important functional fillers, liquid crystals (LC) are widely used to fabricate various liquid-crystalline polymer composites because they combine the basic properties of conventional polymers with those of LC [18,19,20,21]. For example, Finkelmann et al. have prepared an LC shape memory elastomer, which can be stimulated with photo-illumination by incorporating azo groups into the LC [22]. Additionally, Samit V. Ahir et al. have synthesized a new tri-block copolymer with a large central block composed of main-chain nematic units to retain the director alignment, and the resulting LC copolymers show a significant two-way SME [23]. These previous studies have primarily focused on liquid crystalline elastomers or well-defined LC copolymers. Recently, we have developed a facile and versatile method for the preparation of LC-SMPCs. Several series of LC-SMPU composites with both LC properties and shape memory properties were successfully prepared by mixing various SMPU matrixes with 4-*n*-hexadecyloxybenzoic acid (HOBA) [24,25]. Our recent investigations also demonstrated that supramolecular LC-SMPCs based on 4-octyldecyloxybenzoic acid (OOBA) and the supramolecular SMPUs exhibit triple-SMEs and quadruple-SMEs [26]. It was thus proposed that the LC-SMPCs might be good candidates for many potential applications in the field of artificial intelligence. In order to further understand the LC-SMPCs completely and promote their future applications, the significant effects of HOBA and OOBA mesogens on the glass transition and crystal-melting transition of the soft segments have been reported in the previous HOBA-SMPU and supramolecular OOBA-SMPU systems. However, the effect of LC mesogens on the hard segment of SMPU remains unclear. We would like to present a series of investigations about the LC-SMPCs with different LC mesogens in this study.

Therefore, a new kind of LC-SMPCs, termed SMPU-OOBAm, were prepared with PEG-based SMPU containing about 40 wt % hard segment as the polymer matrix, and OOBA mesogen as the LC fillers. This study investigates the effect of OOBA on the structure, morphology, and properties of the SMPU-OOBAm. Notably different from previous systems, the present LC-SMPCs show a successful combination of the liquid crystalline properties, triple-shape memory effects, and self-healing properties. In particular, the self-healing mechanism may be notably different from the common self-healing materials. Thus, this study may be useful in order to fundamentally examine the multi-functional properties, multi-responsiveness, and efficient actuating method of SMPs and polymer composites.

## 2. Experimental Section

### 2.1. Materials

Extra pure grade polyethylene glycol (M_n_ = 6000, PEG6000), was dried before usage at 80 °C under 0.1–0.2 MPa for 6 h. 4,4-Diphenylmethane diisocyanate (MDI, analytical grade), 1,4-butanediol (BDO, analytical grade), 4-octocyloxy benzoic acid (OOBA, analytical grade) and dimethylformamide (DMF, high performance liquid chromatography grade, solvent) were purchased from Aladdin Chemical Reagent Co., Ltd. (Shanghai, China) and were used without further purification.

### 2.2. Synthesis of PEG-Based SMPUs

**Scheme 1:**
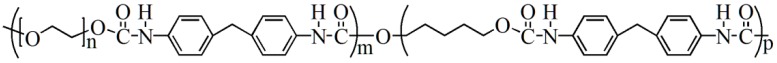
Chemical structure of PEG-based SMPU.

According to our previous work [27], the PEG-based SMPU with 40 wt % hard segment content was easily synthesized by a bulk polymerization method with MDI, BDO, and PEG6000. The chemical structure of PEG-based SMPU is shown in Scheme 1.

### 2.3. Preparation of SMPU-OOBAm Composites

Based on the compositions shown in Table 1, a certain quantity of OOBA was added to the SMPU/DMF solution containing approximately 3.0 g of PEG-based SMPU resin. Under strong mechanical stirring at 80 °C for 2 h, SMPU and OOBA were sufficiently mixed to obtain a homogenous solution-phase mixture. Then, the SMPU-OOBAm composites for the following tests were obtained by casting the mixture onto a Teflon pan, which was placed at 80 °C for 24 h and further dried at 80 °C for 24 h. The samples were coded as P1–P5 (SMPU-OOBAm, m = 0.2, 0.4, 0.6, 0.8, and 1.0, which is the molar ratio of OOBA/MDI).

### 2.4. Materials Characterization

Fourier transformation infrared (FT-IR) spectra were scanned from smooth polymer films with a thickness of 0.2 mm using a Nicolet 6700 FT-IR spectrometer (Nicolet, Madison, WI, USA) by the FT-IR attenuated total reflection (ATR) method. Ten scans at 4 cm^−1^ resolution were signal averaged and stored as data files for further analysis.

The surface morphology of the samples was examined using scanning electron microscopy (SEM, SU-70, Hitachi, Tokyo, Japan), 20 kV, equipped with an energy dispersive X-ray detector for elemental composition. Prior to scanning, the samples were coated with a thin layer of gold. Fraction morphology was scanned after the samples were fractured with liquid nitrogen.

X-ray diffraction (XRD) experiments were performed on a BRUKER AXS D8 Advance diffractometer (BRUKER-AXS, Karlsruhe, Germany) with a 40 kV FL tube as the X-ray source (Cu Kα) and a Lynxeye-Xe detector. The applied voltage is 40 kV. Film samples were used in this experiment.

Thermo Gravimetric Analyzer (TGA) spectra were recorded on a computer-controlled TG Q50 system (TA, New Castle, PA, USA) after drying at 100 °C, under the following operational conditions: heating rate 10 °C/min, temperature range 100–600 °C, sample weight about 5.0 mg, using the film sample in platinum crucibles, 60 mL/min N_2_ flow.

Differential scanning calorimetry (DSC) measurements were carried out with a TA Q200 (TA, New Castle, PA, USA) instrument having nitrogen as the purged gas. Indium and zinc standards were used for calibration. Both heating scan rate and cooling. The scan rate is 10 °C/min. The second heating curves were used for analysis.

Dynamic mechanical analysis (DMA) curves of the samples were determined using a Perkin-Elmer DMA (TA, New Castle, PA, USA) at 1 Hz and at a heating rate of 2 °C/min. The change in modulus and the recovery stress were measured with a DMS module and TMA module, respectively.

Mesophases were identified, and the phase transition temperatures were determined using a Zeiss-Axioscope polarised optical microscope (POM) (Mshot, Guangzhou, China) equipped with a Linkam-THMS-600 variable-temperature stage at a scan rate of 2 °C/min. The samples were heated from 25 to 160 °C and cooled from 160 to 25 °C.

Thermally induced triple-shape memory behaviours were examined via thermo-mechanical analysis using a TA Instruments DMA Q800 system (TA, New Castle, PA, USA) with tension clamps in controlled-force mode. Samples were dried at 100 °C for 24 h and cut into rectangular pieces of 10 mm × 2.0 mm × 0.5 mm.

### 2.5. Characterization of Isothermal Crystallization Kinetics

Isothermal crystallization experiments were performed with a TA-Q200 DSC instrument (TA, New Castle, PA, USA) using nitrogen as the purge gas, according to the procedure described in the literature. The sample (4–6 mg) was initially heated to 200 °C at a rate of 10 °C/min and held for 5 min to remove the thermal history of the crystallizable phase. Subsequently, the sample was rapidly cooled (−60 °C*/*min) to a designated crystallization temperature (*T*_c_) and held at this temperature until the end of the exothermic crystallization. The heat flow during the isothermal crystallization process was recorded as a function of time. *T*_c_ was chosen as 137 °C in this experiment. The amounts of heat generated during the development of the crystal phase were recorded and analysed according to the typical equation used for evaluating the degree of relative crystallinity (*X*_t_):

(1) $$X_{t}=\frac{\int_{t0}^{t}\left(\frac{dH}{dt}\right)dt}{\int_{t0}^{t=\propto }\left(\frac{dH}{dt}\right)dt}$$

where *t*_0_ and *t* = ∞ are the times at which the sample reached isothermal conditions (as indicated by a flat baseline after an initial spike in the thermal curve) and the time at which the dominant sharp exothermic peak ends, respectively. *H* is the enthalpy of crystallization at time *t*. After isothermal crystallization, the sample was heated to 200 °C, and the crystal melting temperature (*T*_m_), indicated by the maximum of the endothermic peak, was recorded. 

The crystallization kinetics were analysed according to the modified Avrami equation, known as the Ozawa equation:

(2) $$X(t)=1-\exp(-Kt^{n})$$

Equation (2) can be converted into the following form:

(3) log{−ln(1−X(t))}=nlogt+logK

A plot of log{−ln(1 − *X*(*t*))} against log(*t*) should yield a straight line. By fitting the lines, *n* and log(*K*) can be calculated from the slope and intercept, respectively.

Additionally, the half-crystallization time (*t*(0.5)) is defined as the time at which the crystallinity is equal to 50%, and is related to the Avrami parameter *K* as determined with the following expression:

(4) $$K=\ln2/{\left(t(0.5)\right)}^{n}$$

## 3. Results and Discussion

### 3.1. Structural Analysis

**Figure 1:**
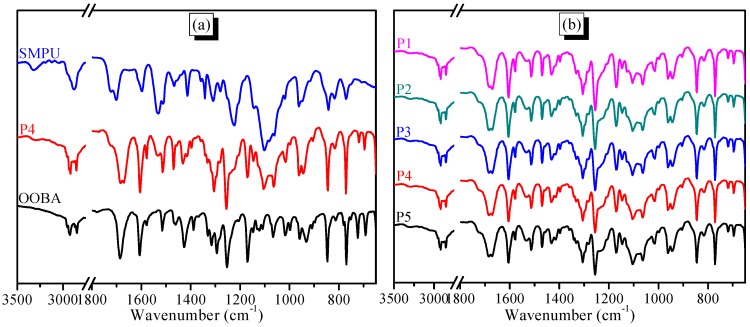
FT-IR spectra of (**a**) sample P4, SMPU, and OOBA; (**b**) SMPU-OOBAm composites with different OOBA content, e.g., P1, P2, P3, P4, P5.

Figure 1 shows the FT-IR spectra of SMPU, OOBA, and SMPU-OOBAm composites (e.g., P1, P2, P3, P4, P5). In the spectrum of OOBA (Figure 1a), the C=O stretching vibration was detected at approximately 1683 cm^−1^, which suggest the formation of hydrogen bond associations. Thus, OOBA molecules may form dimers and create a long lath-like structure with a three-ring core, which is the basic structure of mesogen, to form the LC phase. In the spectrum of SMPU, the N–H stretching vibration is detected at approximately 3323 cm^−1^ and 3286 cm^−1^, whereas the C–N stretching vibration occurs at 1533 cm^−1^ and 1531 cm^−1^. In the SMPU-OOBAm composites (e.g., P4), the N–H stretching vibration shifts to a low wavenumber, which implies the formation of a hydrogen bond between the OOBA and the hard segment of SMPU. Additionally, P4 shows a new frequency at approximately 1685 cm^−1^, which can be ascribed to the C=O stretching vibration of OOBA. The frequency at approximately 1166 cm^−1^ also likely results from the C–O stretching vibration of OOBA. These results suggest that OOBA has successfully incorporated into SMPU and has formed an SMPU-OOBAm composite. Figure 1b also shows that all SMPU-OOBAm composites have similar FT-IR results to P4. The peak density at 1685 cm^−1^ obviously increases from P1 to P5, which confirms the increased OOBA content. In the previous literature, the LC phase was lost when LC mesogens containing pyridine moieties were attached to the SMPU containing carboxyl groups through hydrogen bonding between pyridine and COOH [24]. However, in this work, the SMPU-OOBAm composites tend to keep the intrinsic LC properties of OOBA because of their dimer structure. The first advantage of the SMPU-OOBAm composites is that the liquid crystalline properties of OOBA can be used in the form of polymeric materials for many applications, being more conducive to the applications of liquid crystal materials.

### 3.2. Morphology Analysis

**Figure 2:**
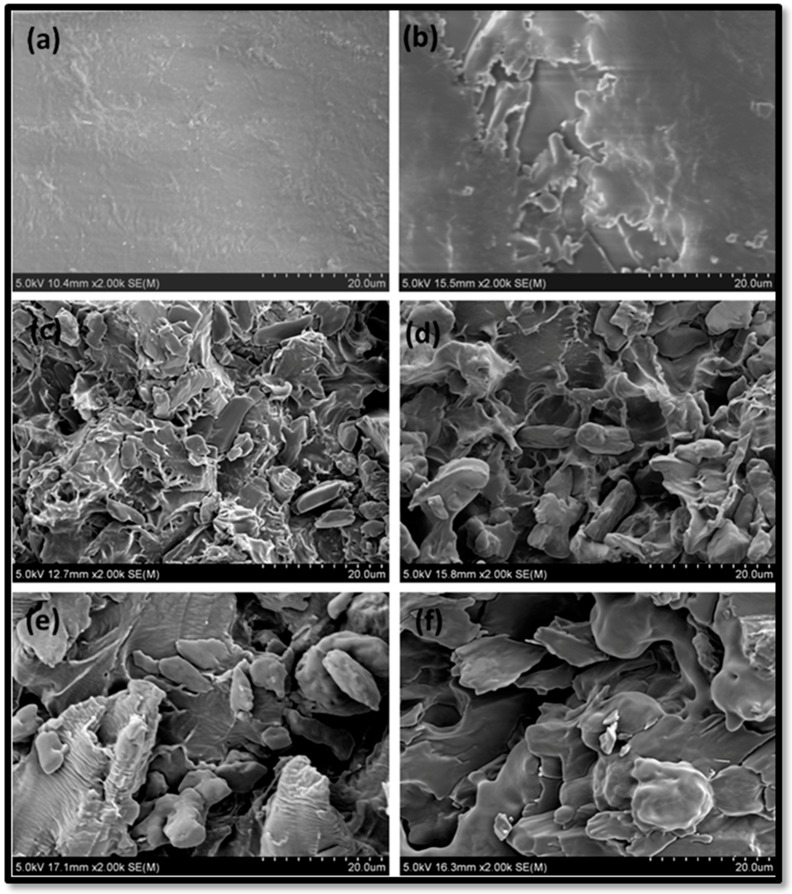
SEM images of (**a**) SMPU; (**b**) P1; (**c**) P2; (**d**) P3; (**e**) P4; and (**f**) P5.

The surface morphology of the SMPU-OOBAm composites was investigated systematically via SEM and XRD. As shown in Figure 2, the SEM images of the pure SMPU exhibit a smooth surface with no hole, but the surface becomes rough after OOBA is incorporated into SMPU (Figure 2b–d). When the OOBA-content increases, more cracks are observed. These broken surfaces demonstrate that the SMPU-OOBAm forms a two-phase separated structure (Figure 2c,d): the OOBA phase and the SMPU matrix. For example, for P5, some self-assembled crystals are observed, which the free OOBA molecules form, but the other OOBA molecules could not be precisely estimated because they are wrapped into a notably small size by the SMPU matrix. It has been confirmed that the incorporated OOBA enters the polyurethane matrix and self-assembles into the free OOBA phase in the composites. Unlike the previous SMPU-HOBA systems, OOBA does not form individual lamellar crystals in the SMPU-OOBAm composites, and the OOBA molecules have better dispersion in the SMPU matrix. This might affect the structure, morphology, and properties of the SMPU greatly.

**Figure 3:**
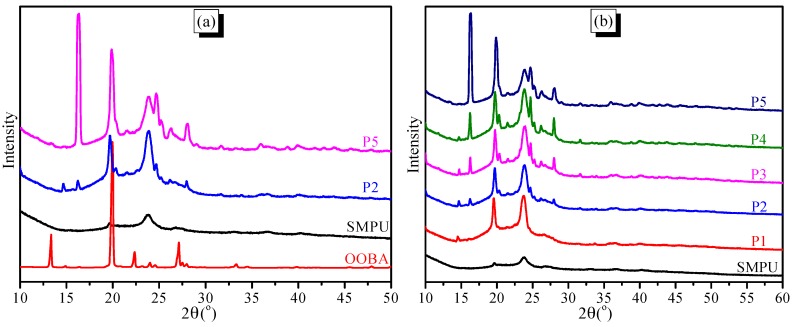
WAXD patterns of the samples (**a**) OOBA, SMPU, P2 and P5; (**b**) SMPU and SMPU-OOBAm composites with various OOBA contents.

Wide-angle X-ray diffraction (WAXD) experiments were used to further study the morphology of the SMPU-OOBAm composites. As shown in Figure 3a, only two weak, broad peaks are observed in the pure SMPU at 19.5° and 23.8°, assigned to the (120) and (132) crystal faces, represents the crystal of polyethylene glycol, which indicates that the pure SMPU contains only a semi-crystalline soft phase and an amorphous hard phase. In addition, the pure OOBA shows many sharp peaks in the range of 10°~35°, which indicates a complete crystalline structure of OOBA [28,29]. When the OOBA is incorporated into the SMPU, nearly all crystalline peaks of OOBA remain detected, e.g., P2 and P5 (see Figure 3a), and the intensity of these crystalline peaks increases when the OOBA content increases (see Figure 3b). The crystalline peaks of the PEG soft phase are also intensified with the increase in OOBA content. This result indicates that OOBA promotes the crystallinity of the soft phase of SMPU. However, the intensity of the crystalline peaks in SMPU-OOBAm is not as strong as that of pure OOBA, which implies a reduced crystallinity of OOBA. Moreover, a new peak at 16.2° is observed; which also intensifies with an increase of OOBA-content. According to the literature [30], we know that pure OOBA can form two different crystals (*α* and *β* crystalline phases) at low temperature. Therefore, this new peak may be ascribed to the second crystal type (*β* phase) of OOBA. Thus, the crystallizability of the first crystal type (*α* phase) of OOBA is restrained, and OOBA subsequently tends to form a second *β* phase when the OOBA content increases. This phenomenon can also be reflected in the thermal properties and phase transitions.

### 3.3. Thermal Properties

**Figure 4:**
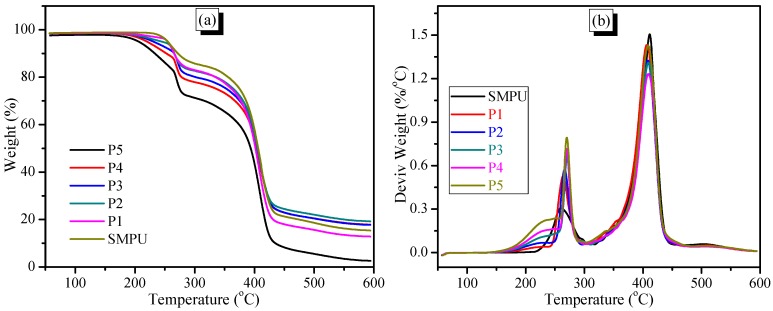
TG-DTG curves of the samples. (**a**) TG curves and (**b**) DTG curves.

The thermal properties of the SMPU-OOBAm composites were investigated by TGA and DSC. The TG curves show that all samples appear to decompose in two stages, which corresponds to the decomposition of the soft (at 200–300 °C) and hard segments (at 350–450 °C) of the SMPU matrix (see Figure 4a). The TG results also show that the decomposition temperatures of the SMPU-OOBAm composites increase when the OOBA-content increases, which implies that the decomposition of SMPU-OOBAm is restrained when OOBA is incorporated. However, when the OOBA-content increases, the decomposition quantity in the first stage increases because of the increased decomposition of OOBA. Additionally, a broad weak peak is observed at about 210 °C on the DTG curves. This result demonstrates that the thermal stability of the composites at the first stage improves (see Figure 4b). The improved thermal stability make the composites good polymeric materials.

**Figure 5:**
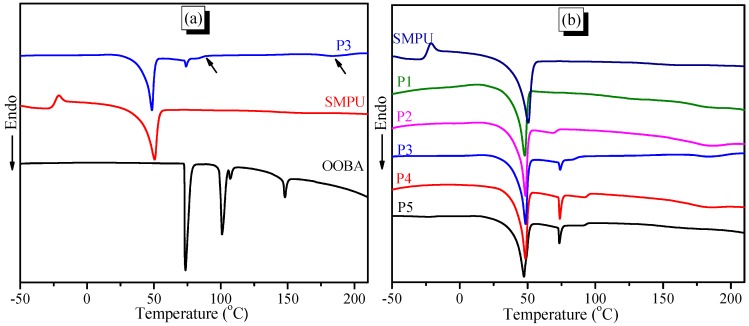

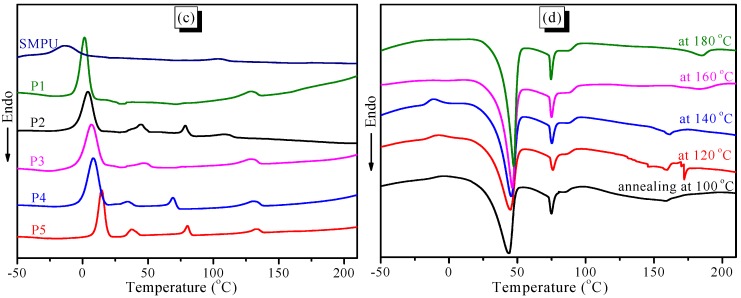
DSC curves of samples (**a**) OOBA, SMPU, and P3 on the second heating process; (**b**) SMPU-OOBAm composites on the second heating process; (**c**) SMPU-OOBAm composites on the cooling process; (**d**) P3 thermal treated at various annealing temperatures.

The second DSC heating curves demonstrate that the pure SMPU shows an exothermic peak at −20 °C and an obvious endothermic peak at 50 °C (see Figure 5a), which correspond to the cold crystallization and crystal-melting temperature of the soft phase, respectively. Pure OOBA shows four obvious endothermic peaks (Figure 5a). Among them, the first two peaks at 78 °C and 101 °C can be attributed to the crystal-melting transitions of the *α* and *β* crystalline phases, respectively. The *β* crystal-melting transition also indicates that OOBA enters the smectic phase. The third peak at 107 °C indicates a smectic-nematic phase transition, and the fourth peak at 148 °C suggests a phase transition from the nematic phase to the isotropic phase. When OOBA is incorporated into the SMPU matrix, the two crystal-melting transitions in the composites (e.g., P3) at 49 °C and 74 °C can be attributed to the crystal-melting of the soft segment of SMPU and the *α* phase of OOBA, respectively. Meanwhile, a new weak exothermic peak which can be attributed to the *β* crystal-melting transition appears at 89 °C. When the molar ratio of OOBA/MDI is lower than 0.4 (m ≤ 0.4), the *β* crystal-melting transitions cannot be detected on the second DSC heating curve (see Figure 5b), because the SMPU polymer chain affects the order structure of OOBA. In addition, another weak exothermic peak is detected at 183 °C and is attributed to the crystal melting of the hard segment of SMPU. This result indicates that the crystallizability of the hard segment of SMPU is also enhanced, because the OOBA crystals provide the nuclear seeds of crystallization for the hard phase. However, the cooling curves demonstrate that pure SMPU shows two weak peaks at 105 °C and −14 °C (see Figure 5c), which are the crystallization temperatures of the hard and soft segments of SMPU, respectively; whereas SMPU-OOBAm shows a series of endothermic peaks upon cooling. For P5, the lowest and highest endothermic peaks at 15 °C and 133.6 °C are attributed to the crystallization temperatures of the soft phase and hard phase of SMPU, and these crystallization temperatures are also higher than those of pure SMPU. When the OOBA-content increases, the crystallization temperature of the soft phase of SMPU shifts to a higher temperature range. It is confirmed again that the soft phase of composites with higher OOBA-content has higher crystallinity. In addition, the endothermic peaks at 37 °C should be attributed to the crystallization temperature of the OOBA phase, whereas the endothermic peak at 80 °C shows the phase transition from the isotropic phase to the LC phase. These phase transitions are also verified by the following POM investigations.

The influence of heat treatment was also investigated in this study. In Figure 5d, the effect of OOBA in the composites on the thermal properties during the heat treatment was investigated with DSC by heating sample P3 to 100, 120, 140, 160, and 180 °C. When P3 was thermally treated at 120 °C and 140 °C, the cold crystallization peak of the soft phase of SMPU was detected at −11 °C. However, the peak disappears after the thermal treatment at 100 °C, 160 °C, and 180 °C. A possible reason is that the movement of the polymer chains is frozen at low temperature (<100 °C), and the composites cannot form cold crystallization; the higher treatment temperature (≥160 °C) can promote the movement of the polymer chains and cause a complete crystallization of the soft segments when they cool to a low temperature. Thus, there is no cold crystallization in the second heating process. In addition, Figure 5d demonstrates that the crystallization temperatures of both the soft and hard phases of SMPU shift to higher temperatures when the thermal treatment temperature increases. This result also demonstrates that the heat treatment can promote the crystallizability of the soft and hard segments of SMPU. However, the heat treatment results show that the crystal melting transition of OOBA is maintained at 74 °C in all composites. Thus, OOBA can aggregate into an isolated phase and retain its intrinsic LC properties in the SMPU-OOBAm composites.

**Figure 6:**
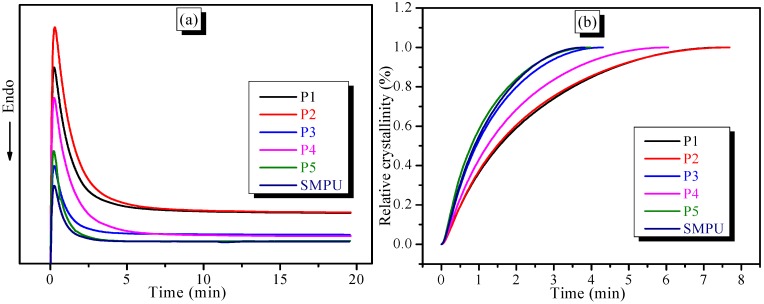

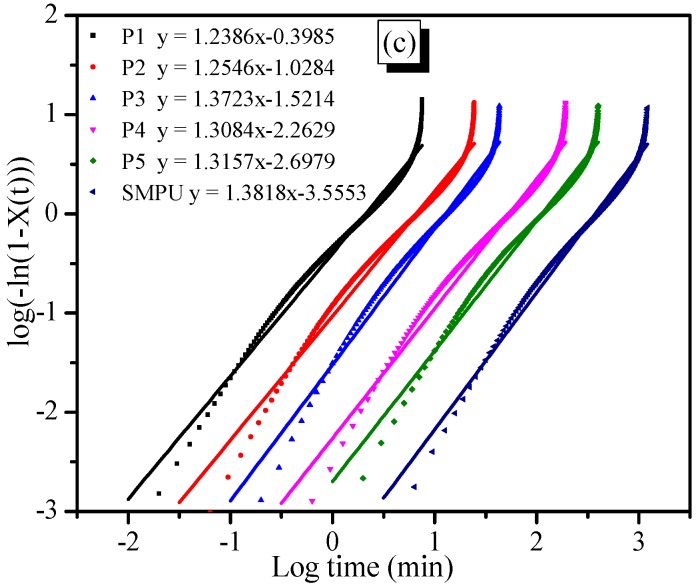
Isothermal crystallization behaviors of the SMPU-OOBAm composites. (**a**) Exothermic curves versus time; (**b**) Dependency of relative crystallinity versus time; (**c**) plots of log{−ln(1 − *X*(*t*))} versus log(*t*) and the fitting line at 137 °C.

For understanding the crystallization behaviors of the hard phase in the SMPU-OOBAm composites, the isothermal crystallization kinetics were investigated using DSC analysis. Detailed calculations are provided in Section 2.5. The crystallization temperature was fixed at 137 °C in this experiment. Figure 6a shows that the exothermic peak of SMPU-OOBAm widens when the OOBA-content increases during the isothermal crystallization process, which suggests the increased crystallization time. According to Equation (1), the relationships between relative crystallinity *(X*_t_) and time are shown in Figure 6b. The curves exhibit an S pattern. The crystallization rate of the hard phase is slow during the initial stage and during the later period of crystallization. Moreover, the slope of the curves decreases when the OOBA-content increases. Thus, the crystallization rate of the hard phase decreases when the OOBA-content increases. The Avrami equation (Equation (2)) was used to denote the complete crystallization behaviours of the SMPU-OOBAm composites. Generally, and for convenient analysis, the Avrami equation is converted to Equation (3) via linearization. Figure 6c presents the dependence of log{−ln(1 − *X*(*t*))} on log(*t*). It was found that log{−ln(1 − *X*(*t*))} is linearly related to log(*t*), but the curve slightly deviates during the later period of crystallization. This deviation implies the existence of a secondary crystallization, which should be attributed to the phase transformation from sphero-crystal to lamella-crystal. Finally, based on the original data and fitting a straight line, the kinetic parameters *n*, *K*, and *t*_1/2_ of the isothermal crystallization were easily calculated. The results are summarized in Table 2. The *n* value of all samples is ~1.3, which further indicates that the crystallization mechanism of the hard segment of SMPU in all composites is the nucleated mechanism. The crystallization rate decreases because the *K* value decreases when the OOBA-content increases. One possible reason for this is that the increased OOBA content might affect the aggregation of the hard segment of SMPU, though they provide more crystal seeds.

### 3.4. Dynamical Mechanical Properties

**Figure 7:**
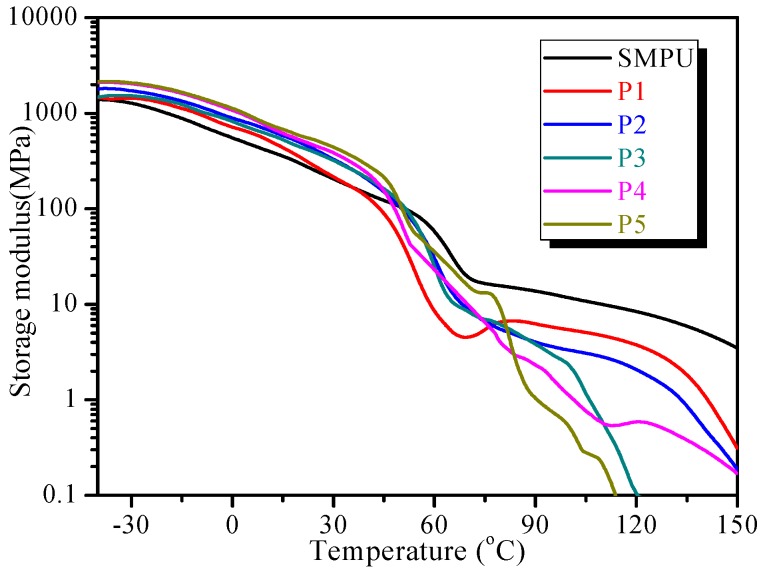
Storage modulus of SMPU-OOBAm composites.

Dynamical mechanical properties can also be used to investigate phase transitions by characterizing the changes of the storage modulus. Figure 7 shows the DMA curves of SMPU-OOBAm composites. All of the samples have a large glassy modulus (*E*’_g_) below −30 °C, whereas the storage modulus gradually decreases because of the glass transition of the soft segment of SMPU. The first significant storage modulus occurs when the temperature increases to 46 °C, because of the crystal-melting of the soft segment of SMPU. Thereafter, SMPU enters the rubber state. The DMA curves demonstrate that the rubber modulus at 46~60 °C in the SMPU-OOBAm composites tends to be less than those of SMPU. This result again confirms that the OOBA crystals reinforce the crystallinity of the soft segment of SMPU, and act as crystal fillers in the rubber state. The second storage modulus change occurs above 77 °C, particularly in samples with higher OOBA-content. This modulus change should be ascribed to the crystal melting transition of OOBA because it exactly corresponds to the *T*_m_ of OOBA. This observation is also notably consistent with the DSC and POM results. Thus, OOBA is another isolated phase that controls the storage modulus. The OOBA isolated phase provides a second reversible phase to the shape fixation and shape recovery. Therefore, SMPU-OOBAm composites may have a triple-shape memory effect.

### 3.5. Liquid-Crystalline Properties

**Figure 8:**
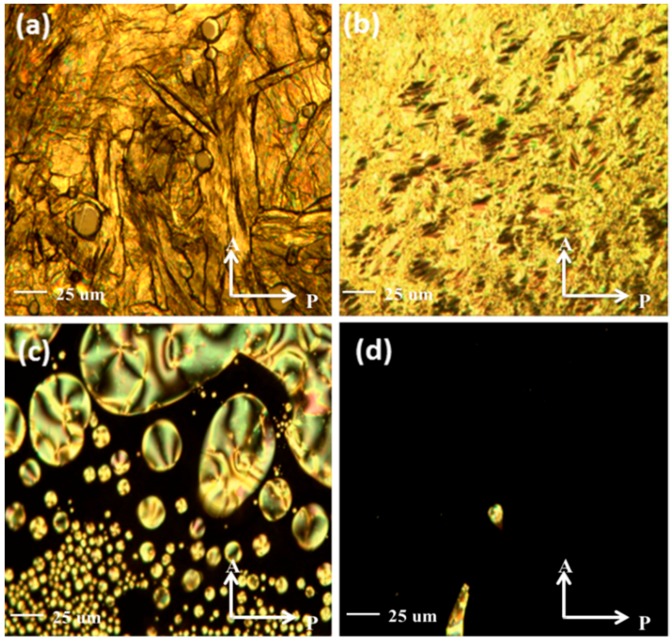
Representative POM images of pure OOBA upon heating (**a**) 50 °C; (**b**) 112 °C; (**c**) 135 °C; and (**d**) 139 °C. (400×) A: analyzer and P: polarizer.

The liquid-crystalline properties and the phase transition behaviors of SMPU-OOBAm composites were investigated by POM. As we known, OOBA exhibits two crystalline forms, followed by smectic and nematic LC phases upon heating. Typical POM textures of OOBA upon heating are shown in Figure 8. At low temperatures, OOBA forms a stable crystalline phase (see Figure 8a). When the temperature increases to 112 °C, the colourful crystalline texture turns into a smectic LC phase (see Figure 8b). When the temperature continuously increases, the smectic LC texture converts to the nematic LC phase at 135 °C (see Figure 8c). As is known, an amorphous substance has no birefringence, and the field of vision is dark. The OOBA has good liquidity in the nematic phase. Thus, it is hard to get a whole continuous nematic texture at 135 °C. Therefore, the dark areas seen in Figure 8c resulted from either the isotropic phase of part of the OOBA or the amorphous substrate. When the temperature reaches 139 °C, the LC texture begins to disappear and the field of vision becomes dark (Figure 8d). This is the isotropic temperature of OOBA, which exactly corresponds to the exothermic peak on the DSC curve (see Figure 5a).

**Figure 9:**
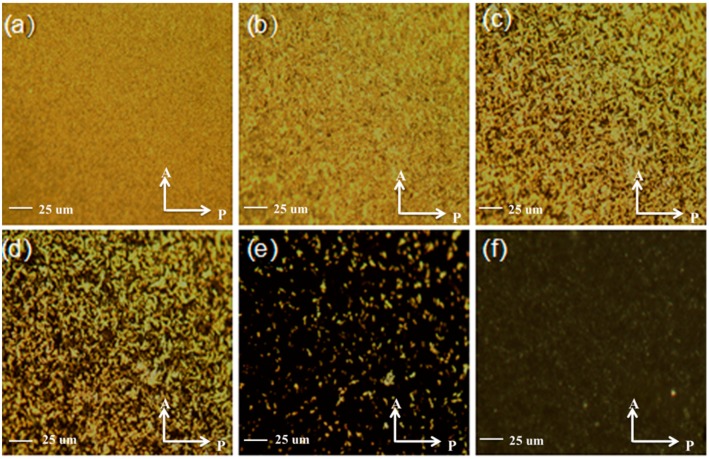
Representative POM images of P5 in the heating process ((**a**) 28 °C; (**b**) 59 °C; (**c**) 76 °C; (**d**) 95 °C; (**e**) 106 °C; (**f**) 180 °C). (400×) A: analyzer and P: polarizer.

For the SMPU-OOBAm composites, sample P5 has a bright abundant crystalline texture at 28 °C (Figure 9a) because the crystals of the soft segment of SMPU and OOBA mixed together. When the temperature increases to over 50 °C, part of the bright crystalline texture disappears (see Figure 9b), and the field of vision becomes darker, which suggests the crystal melting of the soft segment of SMPU. When the temperature reaches 76 °C, the composites enter the smectic phase (see Figure 9c). This result is consistent with the DSC curve (see Figure 5b). At 95 °C, the POM image shows the nematic phase, which suggests the phase transition from smectic phase to nematic phase because of the OOBA. When the temperature reaches 106 °C (see Figure 9e), the nematic phase gradually disappears. Compared with pure OOBA, the LC phase transition temperatures shift to lower temperatures in all composites. These results confirm that the aggregation of OOBA mesogen was restrained by the SMPU matrix, which significantly affected the LC phase transitions. In addition, the POM images also demonstrate that some weak crystalline texture is maintained above 180 °C (see Figure 9f), which implies the crystal formation of the hard segment of SMPU. These phenomena are also identified on the DSC curves.

**Figure 10:**
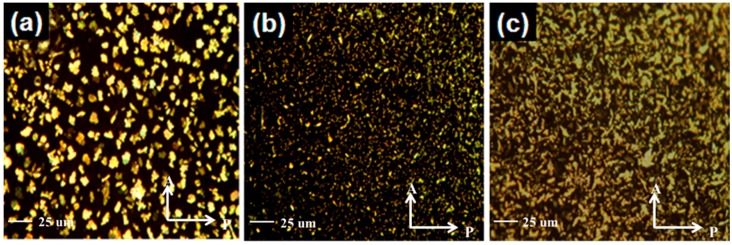
POM images of (**a**) P2 at 86 °C; (**b**) P4 at 95 °C; (**c**) P5 at 95 °C. (400×) A: analyzer and P: polarizer.

Additionally, a comparison study of the POM textures of the composites is shown in Figure 10. The results demonstrate that all composites showed similar LC phase transitions with relatively lower temperatures than pure OOBA. In the composites with lower OOBA contents (e.g., P2 and P4), the OOBA phase tended to disperse into the isolated OOBA phase in an SMPU matrix. As shown in Figure 9, the black phase is the SMPU phase, and the bright phase is the OOBA LC phase. When the OOBA-content was higher, the OOBA phase could also form a continuous OOBA phase in the composites. This result confirms that the crystalline behaviours and LC properties of OOBA are maintained in all SMPU-OOBAm composites. It is thus confirmed again that SMPU-OOBAm composites can make the OOBA mesogens be used in many polymeric materials applications, including flexible displays, electronic papers, etc.

### 3.6. Shape Memory Properties

**Figure 11:**
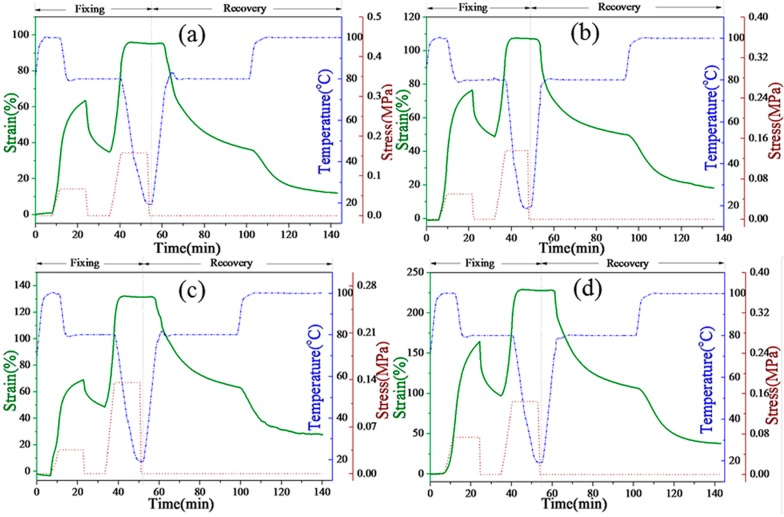
Strain-time-temperature curves showing triple shape memory effects for (**a**) P1; (**b**) P2; (**c**) P3 and (**d**) P4.

Triple-shape memory behaviours of SMPU-OOBAm composites were examined in a thermo-mechanical analysis using a TA Instruments DMA800 with tension clamps in controlled-force mode. In this experiment, the rectangular samples were first elongated at 100 °C, and then fixed at 80 °C, while the second elongation and fixation were carried out at 80 °C and 0 °C, respectively. Thereafter, the two step shape recovery is tested each for 40 min at 80 °C and 100 °C, respectively. The strain deformation, strain fixation, and strain recovery process were recorded for analysis. Figure 11 presents the strain-time-temperature curves of P1, P2, P3, and P4, respectively. The PEG-based SMPU was reported to show only dual-shape memory effects in previous studies. However, Figure 11 demonstrates that all composites show a two-step shape recovery with typical triple-shape memory effects. For example, in sample P1, after 62% strain is deformed at 100 °C, only 38% strain is fixed at 80 °C because of the higher fixation temperature, which is ascribed to the crystallization of the OOBA crystals. However, more than 98% strain fixation is obtained when the temperature decreases to 0 °C because of the further crystallization of the soft phase of the SMPU matrix. During the strain recovery process, P1 recovers 62.6% strain at 80 °C, and the remaining 13.8% strain is quickly recovered when the temperature increases to 100 °C. Finally, more than 90% of the total strain is recovered after the temperature is maintained at 100 °C for approximately 20 min. The strain recovery temperature in the second step corresponds exactly to the crystal-melting temperature of OOBA (76 °C) as mentioned in the DSC and POM results. However, when the OOBA-content increases, the strain recovery ratio in the second step tends to reduce from 90% (P1), 79% (P2), and 78% (P3) to 77% (P4). One possible reason for this is that OOBA has lubricated the SMPU polymer chains as a plasticizer. Although the crystallizability of the hard segment of SMPU is enhanced, the OOBA molecules in the composites may destroy the entire network structure of SMPU, which results in permanent deformation. The shape recovery thus decreases as the OOBA-content increases. However, all composites show good triple-SME. Both the strain fixation and strain recovery are programmable. Therefore, the SMPU-OOBAm can potentially be used in smart optical devices, electronic devices, and sensors.

### 3.7. Self-Healing Properties

**Figure 12:**
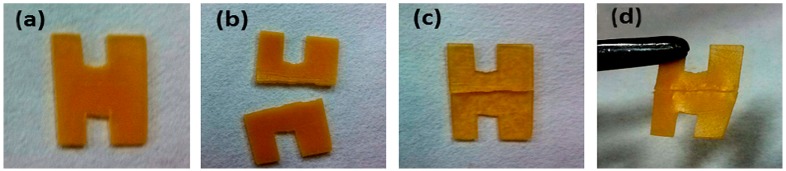
Thermal-induced self-healing process of sample P2 (**a**) the original shape; (**b**) the cut strip; (**c**) the pieced strip after heating; (**d**) the self-healed strip.

The SMPU-OOBAm composites were also found to show self-healing properties in this work. As shown in Figure 12, a specimen with a H pattern was prepared using sample P2. First, the sample was cut into two parts before testing (Figure 12), which were subsequently placed together. When the temperature was increased to 80 °C for 10 min, the sample became soft because of the crystal-melting of the soft segment of SMPU. When heated to 100 °C, the gap between the two cut parts gradually decreased. When the temperature reached 130 °C and the samples had been treated for 10 min, the two cut parts joined together completely (see Figure 12c). After cooling to room temperature, the healed sample was obtained (see Figure 12d). Thus, SMPU-OOBAm exhibits thermal-induced self-healing properties. According to the described structure and morphological analysis, the self-healing process is a thermal-induced self-healing mechanism, which can be explained as follows: the OOBA forms an isolated phase, and the SMPU matrix provides the backbone. When the temperature increases to 100 °C, the crystalline OOBA enters the LC state, and a good fluidity is obtained in OOBA molecules at 130 °C. Thus, the OOBA molecules bleed from the SMPU matrix and spontaneously enter the gap. After cooling to room temperature, the OOBA molecules in the gap form crystals via the crystallization process. Therefore, the gap between two cut parts can be sealed. Therefore, the SMPU-OOBAm composites might also have many promise self-healing applications in the fields of flexible printed circuit, self-protecting textiles, and self-healing concrete.

## 4. Conclusions

This paper describes a new type of LC-SMPU composites, which were prepared with PEG-based SMPU and OOBA mesogens. The effect of OOBA on the structure, morphology, and properties of the material was carefully investigated. The results demonstrate that the LC-SMPU composites have liquid crystalline properties, triple-shape memory properties, and self-healing properties. The incorporated OOBA promotes the crystallizability of both the soft and hard segments of SMPU. However, the isothermal crystallization kinetics revealed that the crystallization rate of the hard segment decreases when the OOBA-content increases. Additionally, the SEM confirmed that the SMPU-OOBAm composites form a two-phase separated structure, which was comprised of an SMPU phase and an OOBA phase. DMA further reveals that the composites show two-step modulus changes upon heating. Therefore, the SMPU-OOBAm composites exhibit triple-shape memory behaviour, but the shape recovery ratio decreases with the increase in OOBA-content. Because of the crystalline behaviours and LC properties of OOBA that are maintained in all SMPU-OOBAm composites, the SMPU-OOBAm composites have good self-healing properties, due to the heating-induced “bleeding” of OOBA in the liquid crystalline state and its subsequent crystallization upon cooling. Therefore, the SMPU-OOBAm composites have promising applications in smart optical devices, smart electronic devices, and smart sensors.

## Figures and Tables

**Table 1:** Composition of SMPU-OOBAm composites.

Sample	SMPU (g)	OOBA (g)	Molar Ratio of OOBA/MDI
Pure SMPU	3	–	–
P1 (SMPU-OOBA0.2)	3	0.1628	0.2
P2 (SMPU-OOBA0.4)	3	0.3256	0.4
P3 (SMPU-OOBA0.6)	3	0.4884	0.6
P4 (SMPU-OOBA0.8)	3	0.6512	0.8
P5 (SMPU-OOBA1.0)	3	0.8140	1.0

**Table 2:** Parameters of the isothermal crystallization kinetics for SMPU-OOBAm composites.

Samples	*n*	ln*K*	*K*	*t* (0.5)
P1	1.2386	−0.3985	0.6713	1.0404
P2	1.2546	−1.0284	0.3576	2.2942
P3	1.3723	−1.5214	0.2184	4.8786
P4	1.3084	−2.2629	0.1040	11.9560
P5	1.3157	−2.2679	0.1035	12.2027
